# Normobaric oxygen therapy increases cartilage survival ratio in auricular composite grafting in rat models

**DOI:** 10.1016/j.jpra.2018.07.003

**Published:** 2018-07-31

**Authors:** Yusuke Hamamoto, Tomohisa Nagasao, Niyazi Aizezi, Motoki Tamai, Tetsukuni Kogure, Tadaaki Morotomi, Noriyuki Tagichi, Yoshio Tanaka

**Affiliations:** a Department of Plastic and Reconstructive Surgery, Faculty of Medicine/Graduate School of Medicine, Kagawa University, Kida County, Miki-Cho Ikenobe 1750-1, Takamatsu, Kagawa, Japan; b Department of Plastic and Reconstructive Surgery, Faculty of Medicine/Graduate School of Medicine, Kindai University, Sayama City, Ono‐higasi 377‐2, Osaka, Japan

**Keywords:** Composite graft, Oxygen, Blood, Cartilage, Ear, Auricle

## Abstract

**Purpose:**

This study aims to clarify whether normobaric oxygen therapy improves the survival of auricular composite grafts in rats.

**Methods:**

For 10 male SD rats, 1.5 cm^2^ composite grafts were harvested from bilateral ear regions including whole auricles. The harvested grafts were transferred caudally and sutured there. The 10 rats were randomly divided into two groups and kept for 21 days in two different circumstances. The first group (Control group: five rats carrying 10 grafts) was kept in room air (20% oxygen) throughout the 21 days, and the second group―named NBO (normobaric oxygen) group (five rats carrying 10 grafts)―was kept in normobaric 60% oxygen for 3 days and then in room air for 18 days. All the 10 rats were sacrificed on the 21st day. Surviving areas of the grafts and the height of the surviving auricular cartilage were examined for statistical comparison of the two groups. Furthermore, the conditions of chondrogenesis occurring around the perichondrium were compared between the two groups.

**Results:**

Surviving areas did not present statistically significant differences between the two groups. The height of surviving cartilage was significantly greater for the NBO group (2610 ± 170 SD µm) than that for the Control group (1720 ± 190 SD µm). Chondrogenesis occurred at positions more distant from the recipient bed in the NBO group than that in the Control group.

**Conclusion:**

Normobaric oxygen therapy increases the thickness of surviving cartilage in auricular composite grafting in rats, thus suggesting that NBO therapy may also be effective in composite grafting for humans.

## Introduction

**Figure 1:**
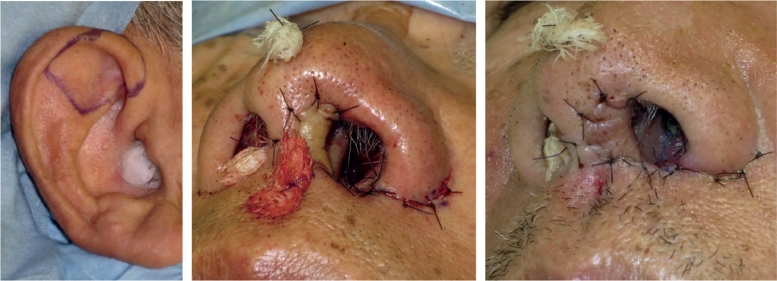
An instance of facial reconstruction with auricular composite graft. A composite graft harvested from the helical crus (Left) was transplanted to a defect of the columella (Center). (Right) The condition on the 7th postoperative day: the graft completely survived.

Free composite grafting is a surgical procedure in which different kinds of combined tissues are transplanted without vascular anastomosis. Plastic surgeons often use auricular composite grafting—consisting of cartilage, subcutaneous fat, and skin―in their clinical practices. The skin of the ear is similar to the skin of the face in texture and color. Furthermore, the inclusion of cartilage allows surgeons to form the graft into desired three-dimensional shapes to fit defects by using cartilage as a framework^1^, ^2^, ^3^, ^4^. Because of these advantages, auricular composite grafting is a workhorse for the reconstruction of the eyelid, columella, and nostril rim (Figure 1).

On the other hand, graft survival is not necessarily reliable in auricular composite grafting. It takes 2–3 days for angiogenesis from the recipient bed into the graft to occur. Furthermore, the supply of oxygen to the graft depends solely on diffusion from the recipient bed to the graft. Hence, parts of the graft distant from the recipient bed cannot receive sufficient oxygen to survive, thus developing necrosis. Thus, oxygen supply regulates the size of the graft that can be safely transplanted.

Therefore, while performing the reconstruction of defects above certain sizes with auricular composite grafting, efforts should be made to maximize the part of the graft capable of survival. Specific examples of clinical trials to improve outcomes by increasing oxygen supply include hyperbaric oxygen (HBO) therapy for diabetic feet^5^ or sudden sensorineural hearing loss.^6^ In animal studies, HBO therapy was reported to increase survival areas of flaps and grafts.^7^, ^8^, ^9^, ^10^

In the present study, we focus on normobaric oxygen (NBO) therapy. NBO therapy—putting patients under high-density oxygen at normal pressure—is conducted to improve metabolic effects by increased oxygen supply. NBO therapy is easier to apply clinically than HBO therapy.^11^, ^12^

When patients are put under NBO circumstances, oxygen supply to auricular grafts is expected to increase. Subsequently, a greater part of the graft is expected to survive. The present study aims to verify this hypothesis by experiments with rat models.

## Materials and Methods

The present study was conducted in accordance with the Declaration of Helsinki, under the approval and supervision of the Institutional Review Board of Kagawa University (Approval Number: 15136).

### Graft Harvest and Transplantation

**Figure 2:**
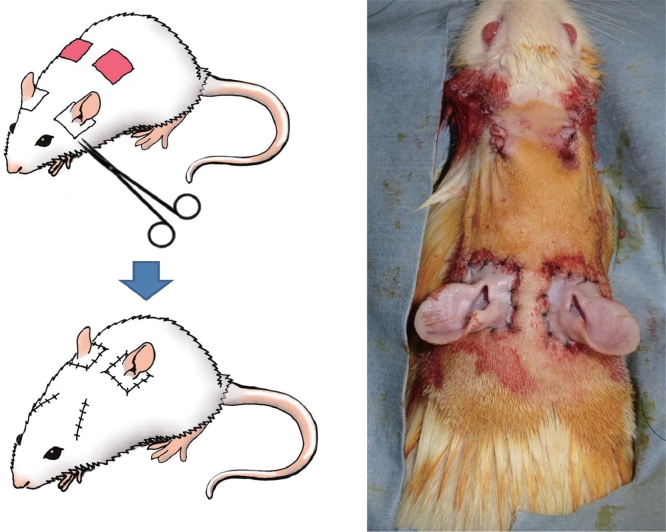
Left: composite grafts were harvested from bilateral auricles and transplanted to the back. (Right) Immediately after transplantation.

Ten male SD rats weighing 300–350 g were included in the study. The rats were anesthetized by intra-abdominal injection of medetomidine hydrochloride (0.3 mg/kg), midazolam (4 mg/kg), and butorphanol tartrate (5 mg/kg). Combined tissues consisting of a square-shaped section of skin and the whole ear were harvested. The length of the square's side was 15 mm. The grafts were removed from the head at the layer of the superficial fascia. Defects were made at bilateral scapular angles of each rat to match the shape and size of the harvested auricular grafts. The harvested grafts were transferred to cover the defects and sutured there with 4-0 Nylon suture (Figure 2).

**Figure 3:**
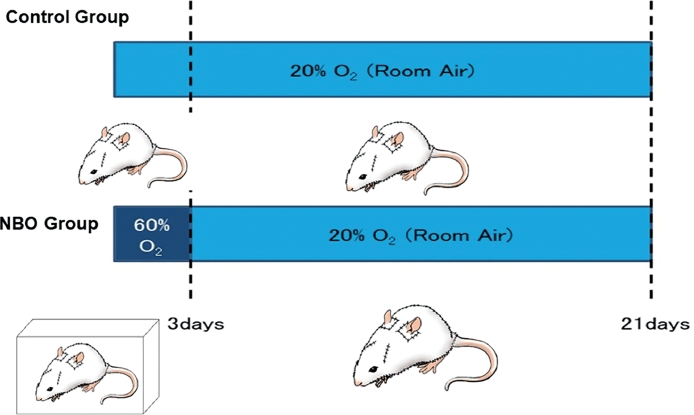
Protocols for oxygen application. Above: in the Control group, the rats were kept in room air for 21 days after operation. Below: in the NBO group, the rats were kept in a box where the density of the oxygen was maintained at 60% for the first 3 days. Then, they were kept in room air for 18 days.

After the operation, the 10 rats were randomly divided into two groups―each consisting of five rats. The two groups were kept in different circumstances. The first group—defined as Control group―was kept in room air for 21 days. The second group was kept in normobaric 60% oxygen for the first 3 days and then in room air for 18 days. The second group was defined as NBO (**N**ormo**b**aric **o**xygen) group (Figure 3). All rats were sacrificed on the 21st day, and the following evaluation was conducted.

### Evaluation

#### Surviving Areas

**Figure 4:**
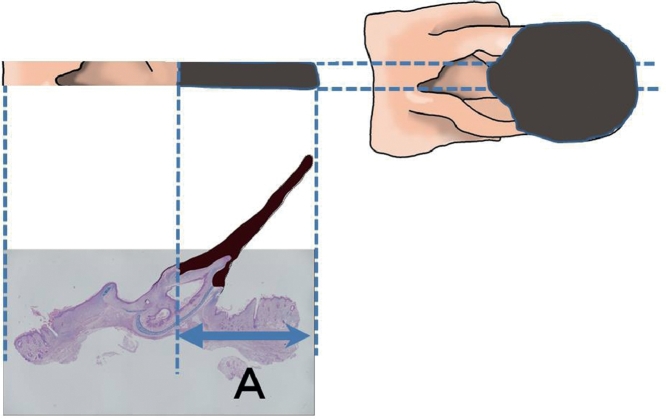
Evaluation of surviving area. The dark-colored zone indicates necrotic tissue. A part was included in the surviving part when its tissue contacting the recipient bed survived. For instance, part A in the figure was included in the surviving area, though the overlying tissue in the part had died.

On the 21st day after surgery, the rats were anesthetized, and photographs of the transplanted composite grafts were taken with a digital camera (Power Shot S120; Canon, Inc., Tokyo, Japan). The areas of surviving parts of the grafts were evaluated with graphics software (Photoshop CC 2018, Adobe Systems, San Jose, California) by counting the number of pixels included in the parts. Survival/necrosis of a part was judged by observing whether the tissues in contact with the recipient bed survived. Even if the overlying tissues of a certain part developed necrosis, the part was included in the surviving area when the underlying tissues―directly in contact with the recipient bed―survived (Figure 4).

#### Height of Surviving Cartilage

**Figure 5:**
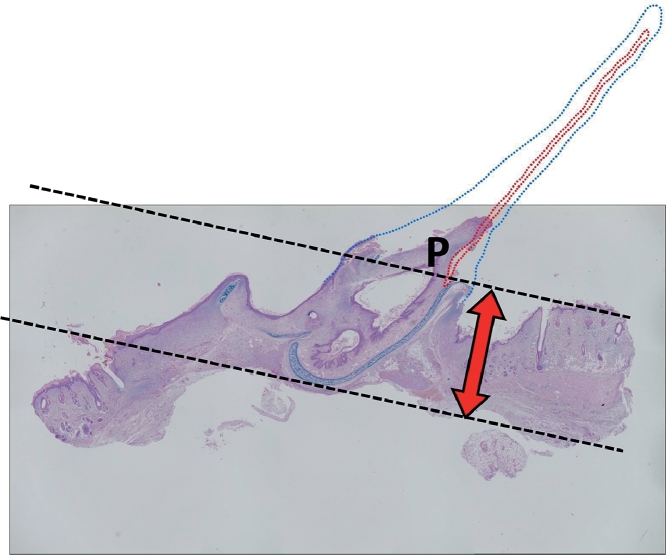
The red and blue dotted lines indicate the initial cartilage and auricular contour. These parts developed necrosis and were removed. P indicates the most distant point of the surviving cartilage from the recipient bed. The distance between the point and the recipient bed was defined as the height of surviving cartilage. (For interpretation of the references to color in the text, the reader is referred to the web version of this article.)

The grafts were stained with Alcian blue and then sliced for histological observation. The as-produced slices were observed with an optical microscope image analysis software system (KEYENCE, Tokyo, Japan). For each graft, the maximum distance of the surviving cartilage from the recipient bed was measured. This distance was defined as the height of surviving cartilage (Figure 5).

#### Chondrogenesis

After staining the harvested grafts with anti-type 2 collagen antibody (Daiichi Chemical, Tokyo, Japan) and Anti-Aggrecan Antibody (cat #.13880-1-AP, Proteintech Japan, Tokyo, JPN), cartilage cells that had generated around the perichondrium were observed and evaluated regarding the three items given below.

### Chondrogenesis Distance from the Recipient Bed

**Figure 6:**
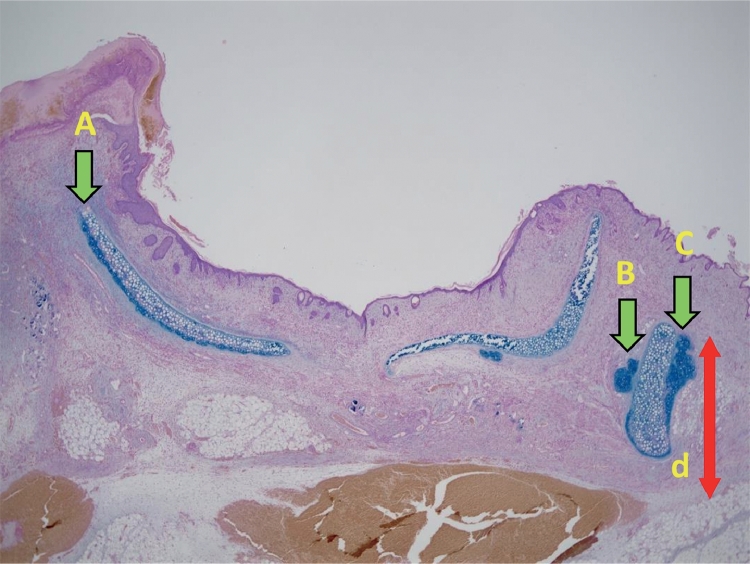
Among the sites at which chondrogenesis occurred (A, B, and C), the point most distant from the recipient bed was identified (C). The distance between the recipient bed and the most distant point (d) was evaluated as the chondrogenesis distance from the recipient bed.

The maximum distance of the point at which chondrogenesis occurred was measured (Figure 6).

### Area of Chondrogenesis

The area of generated cells was measured.

### Location of Chondrogenesis

The locations of chondrogenesis were observed in terms of position between the regenerated chondrocyte and the grafted cartilage. To be more specific, whether regenerated cartilage cells existed above (the side away from the recipient bed) or below (the side facing the graft bed) was observed.

### Statistical Methodology

The above-stated items were compared between the Control (10 grafts in five rats) and NBO (10 grafts in five rats) groups. Statistical analyses were performed with Student's *t* test by using analysis software (GraphPad Prism 5, GraphPad software, San Diego, CA, USA). *P* values equal to or smaller than 0.05 were considered statistically significant.

## Results

### Survival Ratio

**Figure 7:**
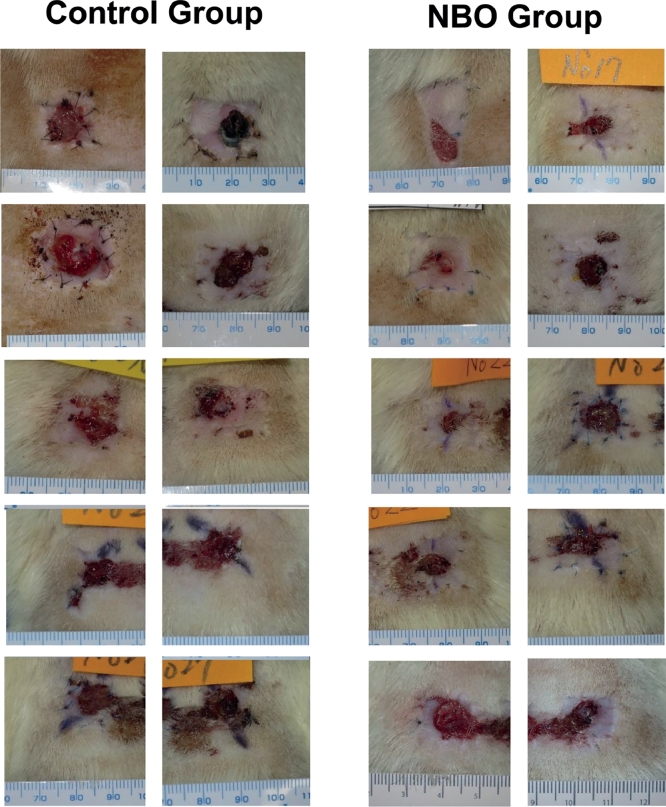
Conditions of grafts on the 21st day.

The photos of the grafts on the 21st day are shown in Figure 7. The survival ratio did not present statistically significant differences between the Control (74.9 ± 7.4 SD%) and NBO (76.1 ± 5.2 SD%) groups (Table 1).

**Table 1:** Graft Survival and Chondrogenesis.

		Control group	NBO group	Statistical significance
Graft survival	Surviving area	74.9 ± 7.4 SD%	76.1 ± 5.2 SD%	NS (*p* = 0.896)
	Height of surviving cartilage	2610 ± 170 SD µm	1720 ± 190 SD µm	*(*p* = 0.0025)
Chondrogenesis	Distance from the recipient bed	1674 ± 276.0	1935 ± 172.4	NS (*p* = 0.2175)
		SD µm	SD µm	
	Area of chondrogenesis	117000 ± 21100 SD (µm^2^)	94100 ± 32400 SD (µm^2^)	NS (*p* = 0.1712)

### Height of Surviving Cartilage

The height of surviving cartilage was significantly greater for the NBO group (2610 ± 170 SD µm) than that for the Control group (1720 ± 190 SD µm) (Table 1).

### Cartilage Regeneration

Chondrogenesis was observed around the perichondrium in six samples of the Control group and seven samples of the NBO group. Statistical comparison of these samples revealed the following findings.

### Chondrogenesis Distance from the Recipient Bed

Chondrogenesis distance from the recipient bed did not present statistically significant differences between the Control group (1670 ± 276.0 SD µm) and the NBO group (1940 ± 172 SD µm) (Table 1).

### Area of Chondrogenesis

Area of chondrogenesis did not present statistically significant differences between the Control group (117000 ± 21100 SD µm^2^) and the NBO group (94100 ± 32400 SD µm^2^) (Table 1).

### Location of Chondrogenesis

**Figure 8:**
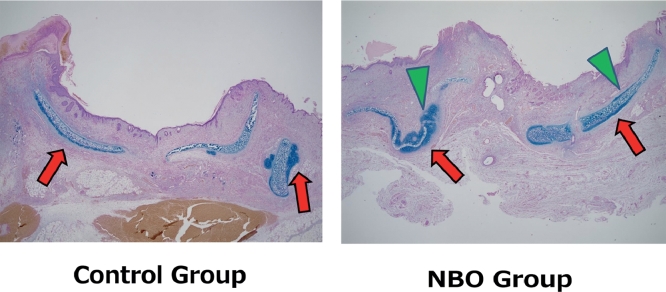
Locational difference of chondrogenesis between the Control (Left) and NBO (Right) groups. Left: in the Control group, chondrogenesis occurred only on the recipient-bed side of the grafted cartilage (Red Arrows). Right: in the NBO group, chondrogenesis occurred on the opposite side (Green Triangular Arrows), as well as on the recipient-bed side. (For interpretation of the references to color in the text, the reader is referred to the web version of this article.)

In six of the 10 samples of the Control group, chondrogenesis occurred only on the recipient bed side. In contrast, in seven of the 10 samples of NBO group, chondrogenesis occurred on the contralateral side as well as the recipient bed side of the cartilage (Figure 8).

## Discussion

Auricular composite grafting is a useful surgical technique for the reconstruction of various defects of the face. However, the survival of the graft is unstable because the supply of oxygen to the graft depends on effusion from the recipient bed in the early stage after transplantation. Though the regions adjacent to the recipient bed can survive safely, those distant from the recipient bed can develop necrosis because of early-stage hypoxia. To achieve optimal results, efforts should be made to maximize the surviving part of the graft. We hypothesized that increasing the supply of oxygen to the graft with NBO therapy may extend the surviving part of the graft, and this was performed in the present study to verify this hypothesis.

In clinical practice, HBO therapy is occasionally used to increase oxygen supply to tissues.^13^ However, the present study spotlights NBO therapy. This is because NBO therapy is easier to apply than HBO therapy. Because special chambers or rooms are indispensable to HBO therapy, it can be conducted only at hospitals where these facilities are available. Moreover, the technical maneuvers of HBO therapy are much more complicated than those for NBO therapy in that HBO therapy requires warming up and cooling down before and after treatment, to avoid decompression sickness and arterial gas embolism.^11^, ^12^ Furthermore, patients cannot wear clothes made of chemical fibers during HBO therapy, because chemical fibers can spark from static electricity and burn. In contrast, NBO therapy does not require special devices and can be performed at ordinary hospitals. Because of these advantages in terms of availability and safety, we put focus on NBO therapy instead of HBO therapy.

In the present study, 60% oxygen was provided for the NBO group for 3 days. This period was defined considering a time-related shift in the mechanism providing oxygen to the graft. After angiogenesis occurs from the recipient beds into transferred grafts, the grafts can receive oxygen from the blood coming through the newly generated vessels. However, before angiogenesis, oxygen supply to the grafts is dependent on the influx from the recipient beds by diffusion. Angiogenesis starts 48 hours after transplantation. Hence, to assure oxygen supply to the graft in the early stage, high-density oxygen needs to be provided at least for 48 hours. In terms of treatment cost, on the other hand, the period of high-density oxygen provision should be minimized. Balancing these effect-versus-cost factors, the rats were placed in 60% oxygen for 3 days in this study. Better survival of the graft might have been achieved if 60% oxygen was provided for more than 3 days, which can be elucidated in future studies.

The findings of the present study are summarized in terms of the following three issues.

First, surviving areas of the grafts showed no statistical differences between the Control and NBO groups. Araki et al.―by studying the effects of NBO therapy on the survival and contracture ratios of composite grafts—reported that NBO therapy increased the survival ratio and decreased the contracture ratio.^14^ Our result is incompatible with their result. Araki used mice; we used rats. The composite grafts in Araki's experiment consisted merely of skin and subdermal fat, whereas the grafts in our study included the whole auricle as well. These differences in experimental conditions might be the cause of the incompatibility. When the whole auricle is transplanted, maintaining its anatomical structure—as we did in this study—only a part of the cartilage contacts the recipient bed. Had we harvested and transplanted the graft in a different way, we might have had a result similar to that obtained by Araki et al. For instance, had we de-epithelialized one side of the auricle and placed it so that the entire de-epithelialized cartilage contacted the recipient bed, greater areas of the grafts might have survived in the NBO group than that in the Control group.

Second, the height of surviving cartilage increased by 1.5 times in the NBO group compared to that in the Control group. According to the previous study by Araki, the partial oxygen pressure of inguinal fat and subcutaneous tissue increased by 195% and 178%, respectively, under 60% oxygen in comparison to that under room air.^14^ Because the recipient beds of our study mainly consist of subdermal fat, the partial oxygen pressure is expected to have also increased by roughly twice in the 60% NBO group. With the increase in the oxygen pressure of the recipient bed, the oxygen pressure of the composite graft increases. Accordingly, the height of the surviving cartilage increased.

Third, chondrogenesis occurred on both sides of the graft (the side facing the recipient bed and its contralateral side) in the NBO group, whereas it occurred only on the recipient bed side in the Control group. We hypothetically explain this phenomenon as follows.

**Figure 9:**
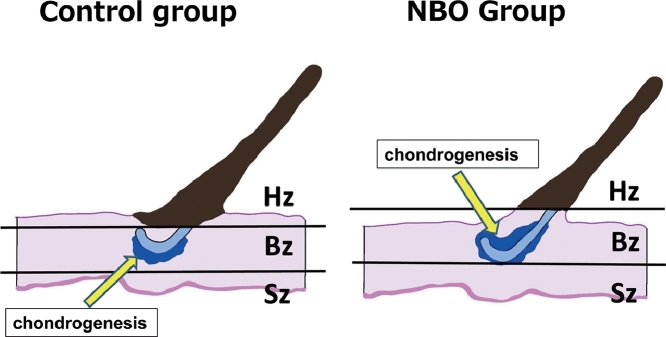
(Left): the regions of the graft were graded into the safe zone (Sz), border zone (Bz), and hypoxic zone (Hz), depending on the partial pressure of oxygen. (Right) NBO treatment makes the safe and border zones shift away from the recipient bed. Accordingly, the position of chondrogenesis—occurring mainly in the border zone—shifts over the cartilage.

In the zone close to the recipient bed, grafted tissues can receive sufficient oxygen supply and survive safely. Conversely, grafted tissues die because of the lack of oxygen in the zone distant from the recipient bed. These zones are temporarily defined as the safe zone and hypoxic zone, respectively. In the zone between these two zones, grafted tissues barely survive because of receiving a minimal amount of oxygen necessary to maintain metabolism. This third zone is defined as the border zone. Gómez-Leduc stated that the stress of ischemia induces the differentiation of mesenchymal stem cells into chondrocytes.^15^^,^^16^ According to this hypothesis, chondrogenesis is likely to occur in the border zone. In the Control group, the border zone exists on the recipient side of the grafted cartilage (Figure 9 Left). Provision of high-density oxygen enables the survival of tissues distant from the recipient bed. Accordingly, the safe zone and the border zone shift away from the recipient bed (Figure 9 Right). Because of this shift, the border zone extends to the contralateral side of the graft, where chondrogenesis occurs, stimulated by the stress of ischemia.

Among the three main findings of the present study, the second finding—the height of surviving cartilage increased by NBO therapy—would appeal to reconstructive surgeons most because of its potential to innovate auricular composite grafting.

Auricular composite grafting is frequently used to reconstruct total or partial thickness defects of the eyelid caused by trauma or tumor removal.^17^ Auricular composite grafting is an effective method in such treatments. However, partial or total necrosis can occur postoperatively, and reoperation is then required using local flaps or other more invasive methods, thus impairing the satisfaction of the patient. Auricular composite grafting is also often used to reconstruct the nasal rim and the columella.^2^^,^^3^^,^^18^^,^^19^ The structural similarity of the auricle to these nasal parts―in that they both consist of cartilage sandwiched by skin—makes auricular composite grafting an ideal technique for nasal reconstruction. However, the graft sometimes develops necrosis, thus negatively affecting patients’ cosmetic quality of life. The second finding implies that these complications can be prevented by performing NBO therapy in postoperative management.

Naturally, the safety of NBO therapy―the problem of potential oxygen toxicity in particular―needs to be carefully considered before using NBO therapy for the given purpose. Our investigation revealed an old report stating that dizziness, tachycardia, and deterioration of vital signs appeared after application of 90% oxygen for 65 hours.^20^ However, 60% oxygen should be much less toxic than 90% oxygen. In fact, the application of oxygen at densities less than 70% is recognized to be nontoxic and is commonly used in the field of intensive care medicine.^21^ Hence, the application of 60% oxygen should carry little risk of oxygen toxicity, though careful monitoring of patients’ conditions is mandatory.

The present study is limited by its relatively small sample size, because survival was evaluated with only 10 composite grafts. If the experiment is repeated with a greater sample size, more clinically meaningful results might be obtained. For instance, although there is no statistically significant difference in the area of chondrogenesis between the Control and NBO groups in the present study―with a *p* value of 0.17―a larger sample size could present a statistically significant difference. Hence, we hope that the present experiment is reproduced with a greater sample size by other researchers.

Nevertheless, the effect of NBO therapy for auricular composite grafting in rats is demonstrated by the present study. We believe that this achievement will allow us to extend postoperative NBO therapy to auricular composite grafting for humans. Under the approval of the institutional review board, we will plan clinical trials in humans in the near future.

## Conflict of interest

None.

## References

[1] Brown JB, Cannon B (1946). Composite free grafts of skin and cartilage from the ear. Surg Gynecol Obstet.

[2] Brown JB, Cannon B (1946). Composite free grafts of two surfaces of skin and cartilage from the ear. Ann Surg.

[3] Lehman JA, Garrett WS, Musgrave RH (1971). Earlobe composite grafts for the correction of nasal defects. Plast Reconstr Surg.

[4] Adams C, Ratner D (2005). Composite and free cartilage grafting. Dermatol Clin.

[5] Chen C-Y, Wu R-W, Hsu M-C, Hsieh C-J, Chou M-C (2017). Adjunctive hyperbaric oxygen therapy for healing of chronic diabetic foot ulcers. J Wound Ostomy Cont Nurs.

[6] Bennett MH1, Trytko B, Jonker B (2012). Hyperbaric oxygen therapy for the adjunctive treatment of traumatic brain injury. Cochrane Database Syst Rev.

[7] Friedman HIF, Fitzmaurice M, Lefaivre JF, Vecchiolla T, Clarke D (2006). An evidence-based appraisal of the use of hyperbaric oxygen on flaps and grafts. Plast Reconstr Surg.

[8] Perrins DJ (1967). Influence of hyperbaric oxygen on the survival of split skin grafts. Lancet.

[9] Zhang F, Cheng C, Gerlach T, Kim DY, Lineaweaver WC, Buncke HJ (1998). Effect of hyperbaric oxygen on survival of the composite ear graft in rats. Ann Plast Surg.

[10] Li EN, Menon NG, Rodriguez ED, Norkunas M, Rosenthal RE, Goldberg NH, Silverman RP (2004). The effect of hyperbaric oxygen therapy on composite graft survival. Ann Plast Surg.

[11] Ambiru S, Furuyama N, Aono M, Otsuka H, Suzuki T, Miyazaki M (2008). Analysis of risk factors associated with complications of hyperbaric oxygen therapy. J Crit Care.

[12] Foster H (1992). Hyperbaric oxygen therapy. J Oral Maxillofac Surg.

[13] Sen CK. (2009). Wound healing essentials: let there be oxygen. Wound Repair Regen.

[14] Araki J, Kato H, Doi K (2014). Application of normobaric hyperoxygenation to an ischemic flap and a composite skin graft. Plast Reconstr Surg Glob Open.

[15] Gómez-Leduc T, Desancé M, Hervieu M (2017). Hypoxia is a critical parameter for chondrogenic differentiation of human umbilical cord blood mesenchymal stem cells in type I/III collagen sponges. Int J Mol Sci.

[16] Huang X, Hou Y, Zhong L (2018). Promoted chondrogenesis of cocultured chondrocytes and mesenchymal stem cells under hypoxia using in-situ forming degradable hydrogel scaffolds. Biomacromolecules.

[17] Chang EI, Esmaeli B, Butler CE (2013). Eyelid reconstruction. Plast Reconstr Surg.

[18] Son D, Kwak M, Yun S, Yeo H, Kim J, Han K (2012). Large auricular chondrocutaneous composite graft for nasal alar and columellar reconstruction. Arch Plast Surg.

[19] Brenner MJ, Moyer JS (2017). Skin and composite grafting techniques in facial reconstruction for skin cancer. Facial Plast Surg Clin North Am.

[20] Becker-Freyseng HCH. (1939). On the question of oxygen poisoning. J Mol Med.

[21] Budinger GRS, Mutlu GM (2013). Balancing the risks and benefits of oxygen therapy in critically ill adults. Chest.

